# Degradation and/or Dissociation of Neurodegenerative Disease-Related Factor Amyloid-β by a Suspension Containing Calcium Hydrogen Carbonate Mesoscopic Crystals

**DOI:** 10.3390/ijms252312761

**Published:** 2024-11-27

**Authors:** Nodoka Iwaya, Akikazu Sakudo, Takuya Kanda, Koichi Furusaki, Rumiko Onishi, Takashi Onodera, Yasuhiro Yoshikawa

**Affiliations:** 1 Faculty of Veterinary Medicine, Okayama University of Science, Imabari 794-8555, Ehime, Japanta-kanda@ous.ac.jp (T.K.); y-yoshikawa@ous.ac.jp (Y.Y.); 2 Mineral Activation Technical Research Center, Omuta 836-0041, Fukuoka, Japan; 3 Santa Mineral Co., Ltd., Minato-ku 105-0013, Tokyo, Japan; 4 Research Center for Food Safety, The University of Tokyo, Bunkyo-ku 113-8657, Tokyo, Japan; atonode@g.ecc.u-tokyo.ac.jp; 5 Environmental Science for Sustainable Development, The University of Tokyo, Bunkyo-ku 113-8657, Tokyo, Japan; 6 Institute of Environmental Microbiology, Kyowa-Kako, Machida 194-0035, Tokyo, Japan

**Keywords:** Aβ, Alzheimer’s disease, amyloid-β, calcium bicarbonate, calcium hydrogen carbonate, Ca(HCO_3_)_2_, disinfection, mesoscopic structure

## Abstract

Amyloid-β (Aβ) aggregates accumulate in the brains of individuals with Alzheimer’s disease and are thought to potentially act as prions, promoting further aggregation. Consequently, the biochemistry of Aβ has emerged as a promising target for Alzheimer’s disease. CAC-717, a suspension of calcium bicarbonate mesoscopic structures derived from natural sources, has been shown to inactivate various pathogens, including prions. This study examined the effects of CAC-717 on both the formation and degradation/dissociation of Aβ aggregates using thioflavin T fluorescence and enzyme-linked immunosorbent assays. Aggregates of Aβ(1–42) peptide were generated by incubation at 37 °C for 24 h, and the effect of introducing CAC-717 on the aggregates was evaluated after further incubation at 25 °C for 30 min. Moreover, CAC-717 was also tested for its ability to inhibit the initial aggregation of Aβ. The results showed that CAC-717 significantly degraded and/or dissociated Aβ aggregates in a concentration-dependent manner. Specifically, CAC-717 treatment for 5 min disrupted Aβ aggregates to give Aβ monomer and oligomer concentrations as high as 130 nM compared to ~10 nM for the water control. In addition, CAC-717 degraded and/or dissociated aggregates within 10 s at 37 °C, and pre-treatment with CAC-717 significantly inhibited aggregation. These results suggest that CAC-717 not only degrades and/or dissociates Aβ aggregates but also inhibits their formation, highlighting its potential as a disinfectant for Alzheimer’s disease.

## 1. Introduction

The brains of individuals affected by Alzheimer’s disease accumulate amyloid-beta (Aβ), a peptide comprising ~40 amino acids [1]. The “Aβ hypothesis” proposes that the accumulation of these Aβ aggregates induces neuronal degeneration, resulting in dementia [1]. The accumulation and aggregation of Aβ play a central role in the pathogenesis of Alzheimer’s disease, which results in neurodegeneration [2]. Aβ deposition occurs early in the disease process and is strongly correlated with cognitive decline [3]. Indeed, the solubility and levels of Aβ peptide in various brain pools are closely related to the disease state [2]. Aβ affects cerebral blood flow and neurovascular coupling [4] and interacts with tau protein at synapses [5]. While Aβ is primarily associated with toxicity, it may also elicit protective effects in neurons [6]. “The amyloid cascade hypothesis” maintains that the major pathogenic mechanism in Alzheimer’s disease is the formation and deposition of Aβ peptide aggregates, which suggests that altered Aβ biochemistry might be a promising target [1]. Indeed, biomarkers related to Aβ have shown potential for both the early diagnosis of Alzheimer’s disease and for monitoring the efficacy of different treatments in clinical trials [7].

It is now well established that prion agents, which induce abnormal protein folding, proliferate in prion diseases [8,9]. The causative agents of other neurodegenerative diseases have been reported to be a type of prion or prion-like factor (prionoid) because they display prion-like behaviors [10,11,12,13]. Moreover, Aβ proteins associated with Alzheimer’s disease may exhibit prion-like transmissibility [10]. Studies have shown that Aβ oligomers can self-propagate and potentially spread throughout the brain [14]. Evidence from iatrogenic cases of Creutzfeldt–Jakob disease suggests possible Aβ transmission through contaminated growth hormone treatments and neurosurgical procedures [15,16]. While not clearly infectious like prion agents, Aβ and other misfolded proteins associated with neurodegenerative diseases demonstrate the ability to spread from cell to cell in the brain [17,18]. These findings raise concerns about the potential transmissibility of protein misfolding diseases [19]. However, there are critical differences between Aβ and prion transmission, particularly in terms of their neurological effects and lethality [20]. The possibility of Aβ transmission remains a subject of ongoing research and debate [21,22,23].

The currently approved treatment for Alzheimer’ disease is Lecanemab, jointly developed by Eisai Co., Ltd. (Tokyo, Japan) and Biogen Inc. (Cambridge, MA, USA), which was granted US Food and Drug Administration approval in 2023. Lecanemab is a humanized IgG1 monoclonal antibody that targets soluble and insoluble aggregates of Aβ [24]. On the other hand, we are interested in naturally derived agents that can inhibit pathological Aβ and detoxify Aβ aggregates via a different type of action.

Calcium bicarbonate, formula Ca(HCO_3_)_2_, is a key nutrient and component of trees [25]. Plant growth points and coral skeletons are rich in natural calcium bicarbonate in the form of mesoscopic structures (nanoparticulate structures of 50–500 nm). A solution of calcium bicarbonate is obtained from mixtures of chrysanthemum, rose, bamboo shoot bark, other plants and coral, which can be slowly broken apart by exposure to ultrasonic vibration [26]. Application of high direct current voltage (8300 V, 100 mA), followed by far infrared radiation (6–14 µm) of the resulting solution leads to the formation of mesoscopic structures of calcium bicarbonate [27]. Previous studies have reported that a suspension prepared by placing the mesoscopic structure crystals in water, termed CAC-717, displays bactericidal and virucidal effects [25,26,27,28,29,30,31]. Furthermore, CAC-717 has an inactivating effect against prions [32], which are considered one of the most resistant forms of pathological agents.

Although CAC-717 may be a promising avenue for enhancing traditional disinfection practices, its mechanism of action remains unknown. CAC-717 has the potential to offer safer and more effective disinfection procedures, particularly in a healthcare setting. Indeed, elucidating the specificity of interaction between CAC-717 and protein aggregates such as Aβ will have wider implications beyond disinfection technology. Here, we have investigated the potential use of CAC-717 as a decontamination agent for neurodegenerative disease-related protein factors by analyzing its effect on Aβ. The aim of the study was to examine the degradation and inhibition of Aβ aggregation by CAC-717, focusing on its potential to redefine disinfection strategies.

## 2. Results

**Figure 1:**
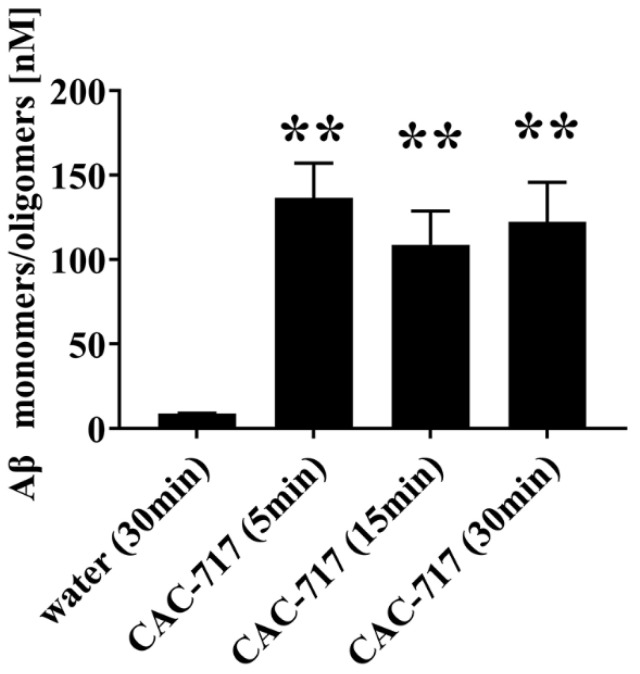
Increase in the concentration of Aβ monomers and oligomers after incubation of Aβ aggregates with CAC-717. Aβ(1–42) was aggregated at 37 °C for 24 h and then treated with CAC-717 for 5, 15 or 30 min. The concentration of Aβ monomers and oligomers in each sample was measured by ELISA. ** *p* < 0.01 compared with water (30 min) by non-repeated measures ANOVA followed with Dunnett’s multiple comparison test.

First, we examined the degrading and/or dissociating effects of CAC-717 on Aβ aggregates. Aggregates of Aβ formed by incubation of 5 µM Aβ(1–42) at 37 °C for 24 h were treated with distilled water or CAC-717 for 5–30 min and then detected with an ELISA to quantify the concentration of Aβ monomers and oligomers. By comparison to distilled water, the amount of Aβ monomers/oligomers that reacted with antibody increased significantly after incubating the Aβ aggregates with CAC-717 for 5, 15 or 30 min (Figure 1). In particular, the concentration of Aβ monomers/oligomers reacting with antibody increased to ~130 nM after only 5 min of treatment with CAC-717, as compared with ~10 nM after treatment with water. This observation suggested that Aβ aggregates were degraded and/or rapidly dissociated following incubation with CAC-717, resulting in a relative increase in the concentration of Aβ monomers and oligomers (comprising 2–30 monomers).

**Figure 2:**
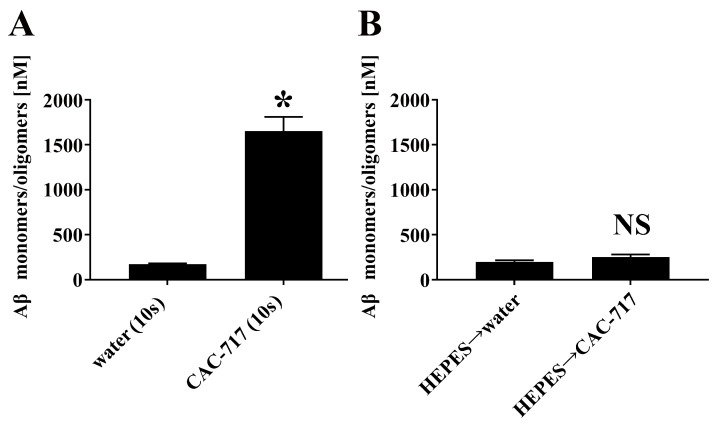
Effects of CAC-717 on the degradation and/or dissociation of Aβ aggregates, and the influence of prior addition of HEPES buffer. (**A**) Aggregated Aβ(1–42), formed by incubating Aβ(1–42) solution at 37 °C for 10 s, was treated with CAC-717 or distilled water for 10 s, and the concentration of Aβ(1–42) monomers/oligomers was measured by ELISA. (**B**) Aggregated Aβ(1–42), formed under the same conditions, was pre-treated with HEPES buffer before exposure to CAC-717 or distilled water for 10 s. The concentration of Aβ(1–42) monomers/oligomers was subsequently measured by ELISA. * *p* < 0.05 compared with water (10 s); NS: no significant difference compared with “HEPES → water” by Mann–Whitney U test.

Next, we tested the effect of CAC-717 treatment on Aβ aggregates obtained by incubation of 5 µM Aβ(1–42) solution at 37 °C for just 10 s. The control experiment using distilled water in place of CAC-717 indicated that the Aβ monomers aggregated to the extent that the Aβ monomer/oligomer concentration was only ~200 nM. However, these Aβ aggregates were degraded and/or dissociated after exposure to CAC-717 for only 10 s, which increased the concentration of Aβ monomers/oligomers to 1500 nM (Figure 2A).

We then tested the effect of adding HEPES buffer in advance to prevent CAC-717 from reacting with the Aβ aggregates. Quantification of Aβ monomers/oligomers by ELISA confirmed that no degradation and/or dissociation by CAC-717 occurred. Specifically, the amount of Aβ monomers/oligomers was ~200 nM in both the “HEPES buffer → distilled water” and “HEPES buffer → CAC-717” conditions, indicating that degradation and/or dissociation of Aβ aggregates by CAC-717 was suppressed by the prior addition of HEPES buffer (Figure 2B).

**Figure 3:**
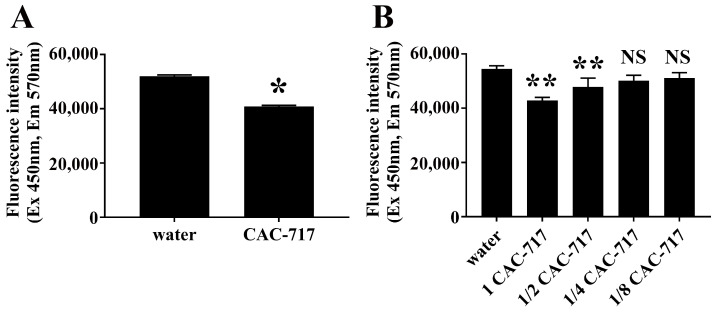
Inhibition of Aβ aggregation by CAC-717 as assessed by thioflavin T fluorescence. (**A**) Decrease in thioflavin T fluorescence upon inhibition of Aβ aggregation by CAC-717. CAC-717 or distilled water was added to a solution of Aβ(1–42) prior to aggregation at 37 °C for 24 h. The extent of aggregation was then examined by fluorescence detection using thioflavin T. * *p* < 0.05 compared with water by Mann–Whitney U test. (**B**) Dose-dependent inhibition of Aβ aggregation by CAC-717. Aβ(1–42) solution was incubated with various concentrations of CAC-717 at 37 °C for 24 h and the extent of aggregation examined by fluorescence detection using thioflavin T. 1 CAC-717, CAC-717 stock solution; 1/2 CAC-717, 2-fold dilution; 1/4 CAC-717, 4-fold dilution; and 1/8 CAC-717: 8-fold dilution. ** *p* < 0.01 compared with water by non-repeated measures ANOVA followed with Dunnett’s multiple comparison test; NS: no significant difference compared with water.

Next, we explored whether CAC-717 can inhibit the aggregation of Aβ(1–42). We measured the fluorescence intensity of thioflavin T in samples of Aβ(1–42) solution that contained CAC-717 during the aggregation process. The presence of CAC-717 in the Aβ aggregation process resulted in lower fluorescence intensity as compared with samples co-incubated with distilled water, indicating that CAC-717 can inhibit the aggregation of Aβ(1–42) peptide (Figure 3A).

To investigate whether the inhibition of Aβ aggregation by CAC-717 is concentration-dependent, we measured the aggregation of Aβ(1–42) peptide co-incubated with a dilution series of CAC-717 (Figure 3B). As indicated by the fluorescence intensity of thioflavin T, the aggregation of Aβ(1–42) was significantly decreased for Aβ(1–42) co-incubated with undiluted CAC-717 and two-fold diluted CAC-717. By contrast, the aggregation of Aβ(1–42) co-incubated with four-fold diluted CAC-717 and eight-fold diluted CAC-717 did not differ from that of Aβ(1–42) co-incubated with distilled water. Thus, the ability of CAC-717 to inhibit aggregation of Aβ(1–42) was concentration-dependent, as was the effect of CAC-717 on the degradation and/or dissociation of Aβ aggregates.

**Figure 4:**
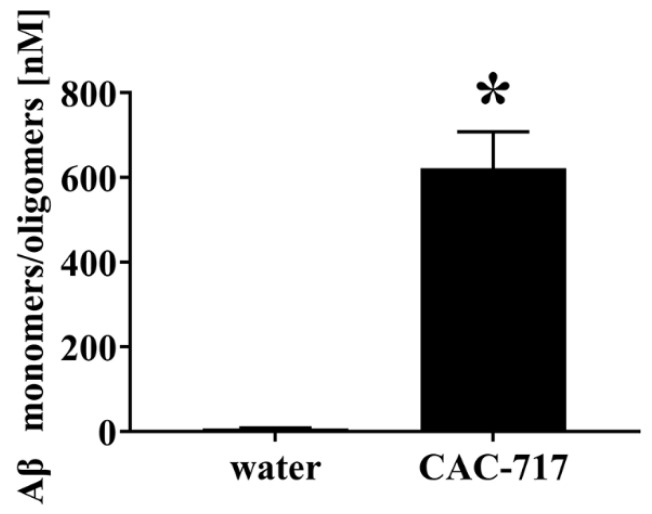
Increase in the concentration of Aβ monomers/oligomers by inhibition of the Aβ aggregation process by CAC-717. CAC-717 or distilled water was added to a solution of Aβ(1–42) before aggregation at 37 °C for 24 h. The resulting concentration of Aβ monomers/oligomers was then measured by ELISA. * *p* < 0.05 compared with water by Mann–Whitney U test.

Lastly, we used ELISA to analyze samples of Aβ after the aggregation inhibition reaction (Figure 4). Following co-incubation with distilled water, a very low concentration of Aβ monomers/oligomers was detected (~10 nM), indicating the formation of many Aβ aggregates. In contrast, the presence of CAC-717 inhibited Aβ(1–42) aggregation as evidenced by the detection of a much higher concentration of Aβ monomers/oligomers (700 nM). These observations suggested that the presence of CAC-717 inhibited the process of Aβ aggregation and simultaneously degraded and/or dissociated Aβ aggregates, resulting in a relative increase in the concentration of Aβ monomers and Aβ oligomers (~2–30 monomers).

## 3. Discussion

Inhibitors of Aβ fibril formation have been extensively studied as potential agents for the prevention and/or management of Alzheimer’s disease. In this regard, several classes of compounds have shown to be effective, including tetracyclic- and carbazole-type molecules [33], apomorphine derivatives [34], hydroxyindole compounds [35], *D*-amino acid peptides [36], benzofurans [37], organofluorine inhibitors [38] and amphipathic molecules such as hexadecyl-*N*-methylpiperidinium bromide [39]. These inhibitors often work by binding to Aβ and preventing fibril formation, sometimes promoting oligomerization instead. Interestingly, some compounds selectively inhibit either oligomerization or fibrillization, suggesting that these processes may be independent [40]. Many inhibitors share common structural features, such as aromatic moieties or specific three-dimensional conformations, that appear to be critical for their efficacy. A better understanding of these inhibitor–peptide interactions will accelerate the development of more potent agents that may act as effective disinfectants for Alzheimer’s disease. In recent years, there has been a drive towards research and development into natural materials that combine potent biological activity with low environmental impact. Here, we focus on the utility of natural mesoscopic structures found in plant growth points and coral skeletons known as CAC-717 [25].

The electrically charged disinfectant CAC-717, which contains mesoscopic calcium bicarbonate crystals, displays striking antimicrobial and virucidal properties [25]. Studies have shown the efficacy of CAC-717 against various microorganisms, including non-enveloped viruses and bacteria, by modifying their genome [28]. CAC-717 has also demonstrated anti-prion activity by reducing both prion transmissibility and the conversion to PrP^Sc^ (abnormal isoform of prion protein) [32]. When combined with sodium dodecyl sulfate, CAC-717 absorbed onto ceramic surfaces also significantly reduced the protein misfolding cyclic amplification seeding activity of scrapie prions and prevented disease development in mouse bioassays [41]. In addition, CAC-717 demonstrated potent virucidal activity against severe acute respiratory syndrome coronavirus 2 (SARS-CoV-2) variants and human noroviruses [29,30,31]. These results suggest that CAC-717 may have potential applications in various disinfection scenarios. Furthermore, because CAC-717 can be used as a disinfectant with less environmental load, it may contribute to a sustainable society, especially in the medical and sanitation fields.

In this study, we extended our work on CAC-717 by examining its effects on Aβ aggregates, the pathological agent in Alzheimer’s disease. Using both thioflavin T fluorescence assays and ELISA, we confirmed the ability of CAC-717 to degrade and/or dissociate Aβ aggregates as well as inhibiting the aggregation of Aβ(1–42). We further showed that the biological activity of CAC-717 was concentration-dependent. Our results allow us to propose the following model for the action of CAC-717 on Aβ. When Aβ(1–42) solution is incubated in the presence of CAC-717, the total number of Aβ(1–42) molecules remains constant, but CAC-717 inhibits the formation of large Aβ(1–42) aggregates while at the same time increasing the ratio of Aβ oligomers (2–30 monomers) and monomers. Based on previous studies, many inhibitors of Aβ fibril formation share key structural features, such as aromatic groups and specific three-dimensional conformations, that are critical for their interaction with Aβ peptides [42]. These features enable the inhibitors to disrupt Aβ aggregation, either by blocking fibril formation or by promoting the generation of smaller, non-toxic oligomers. Given the reported activity of CAC-717 in disrupting prion-like proteins, it is plausible that its mechanism of action on Aβ may involve a similar process. CAC-717 contains mesoscopic calcium bicarbonate crystals that may interact with Aβ aggregates at multiple stages of the aggregation process.

One possible mechanism is that the calcium bicarbonate structure physically interferes with Aβ peptide interactions, leading to fibril destabilization and aggregate dissociation. This process may explain the rapid degradation/dissociation of Aβ aggregates observed in our experiments. In addition, the ability of CAC-717 to inhibit the initial formation of Aβ aggregates suggests that it may interact with Aβ monomers or early-stage oligomers, preventing their alignment into fibril-prone conformations. This behavior is reminiscent of other amphipathic molecules that have been shown to prevent fibrillization by altering the solubility or structural integrity of Aβ peptides. Furthermore, given that the effect of CAC-717 on Aβ aggregation is concentration-dependent, it is possible that its action is mediated by surface interactions between the mesoscopic calcium crystals and Aβ peptides either through ionic interactions or by modulating the local environment around the aggregates. Indeed, the proposed mechanism is consistent with the reported activity of CAC-717 against a variety of pathogens, including prions and viruses [25], where similar structural perturbations may play a role in inactivating target proteins.

Intriguingly, CAC-717 displays dual action in both preventing aggregate formation and disrupting existing aggregates. This dual functionality makes CAC-717 a unique agent with potential applications for disinfecting medical instruments contaminated with amyloidogenic substances, thereby mitigating secondary infections linked to prion-like proteins. CAC-717 is a non-irritant and alkaline solution whose pH rapidly (<1 min) decreases to 8.84 upon contact with skin [27]. According to the Ministry of Health, Labour and Welfare of Japan Guidelines, the biological safety of CAC-717 has been confirmed via an ISO test (ISO 10993-10) [27,43]. In contrast to existing therapies such as Lecanemab, which targets soluble and insoluble Aβ aggregates via an immunological mechanism [24], CAC-717 offers a physical approach to disaggregation that could both complement and expand strategies to combat neurodegenerative disease-related protein aggregates. Nevertheless, despite these promising findings, the study has some limitations, including the in vitro design, which may not fully capture the complex dynamics of Aβ aggregation and disaggregation in vivo. Moreover, further studies using cell culture cytotoxicity assays will be necessary. In future studies, we aim to investigate how the mesoscopic structure of CAC-717 contributes to its mechanism of action as well as examining its in vivo activity.

## 4. Materials and Methods

### 4.1. Reagents for the Formation of Aβ Aggregates

Aβ–protein (human, 1–42; Aβ(1–42); Catalog number 4349-v; Peptide Institute, Inc., Osaka, Japan) was solubilized in phosphate buffered saline (PBS; Takara, Kusatsu, Japan). Aβ oligomers were defined as aggregates of between 2 and 30 Aβ oligomers.

### 4.2. Degradation of Aβ Aggregates by CAC-717

Aβ(1–42) aggregates were formed by incubation of Aβ(1–42) solution (5 μM) at 37 °C for 24 h. An equal volume of distilled water (Otsuka Pharmaceutical Co., Ltd., Tokyo, Japan), CAC-717 (Santa Mineral Co., Ltd., Tokyo, Japan) diluted 2-, 4- or 8-fold with distilled water or a stock solution of CAC-717 (undiluted solution) was then added to the Aβ(1–42) aggregates. After incubation at 25 °C for 30 min, the reaction of CAC-717 was stopped by adding HEPES buffer (4-(2-hydroxyethyl)-1-piperazineethanesulfonic acid; Catalog number 17557-94; Nacalai Tesque, Inc., Kyoto, Japan).

### 4.3. Inhibition of Aβ Aggregation by CAC-717

An equal volume of distilled water (Otsuka Pharmaceutical Co., Ltd.), CAC-717 diluted 2-, 4- or 8-fold in distilled water or a stock solution of CAC-717 (undiluted solution) was added to 10 μM of Aβ(1–42). After incubation at 37 °C for 24 h, the reaction of CAC-717 was stopped by adding HEPES buffer.

### 4.4. Analysis of Aggregates Using Thioflavin T

The fluorescent dye thioflavin T (Catalog number 202-01002; FUJIFILM Wako Pure Chemical Corp., Osaka, Japan) was used to quantify the amount of protein aggregate in samples. The fluorescence properties of the dye vary depending on the surface structure and size of the amyloid fibrils. Thioflavin T was added at a final concentration of 200 μM to Aβ samples in wells of a black microplate (Nunclon 96 Flat Black, Thermo Fisher Scientific, Waltham, MA, USA). The microplate was incubated at 25 °C for 5 min before assessing the extent of Aβ aggregation by measuring the fluorescence intensity (excitation, 450 nm; emission, 570 nm) using a fluorescence plate reader (Spark Control; Tecan, Kawasaki, Japan).

### 4.5. Enzyme-Linked Immunosorbent Assay (ELISA)

An ELISA kit (human/rat β-amyloid (42) ELISA kit WAKO; Catalog number 290-62601; FUJIFILM Wako Pure Chemical Corp.) was used to detect Aβ monomers and Aβ oligomers (comprising ~2–30 monomers). The kit included anti-Aβ antibodies, BNT77 (Catalog number 010-26883; FUJIFILM Wako Pure Chemical Corp.) and BC05 (Catalog number 010-26903; FUJIFILM Wako Pure Chemical Corp.). The measurement plate was coated with BNT77 antibody, which recognizes residues 11–28 of Aβ. Horseradish peroxidase-labeled antibody (BC05), which recognizes the C-terminus of Aβ, was added to the wells to form a solid-phase antibody–antigen-labeled–antibody sandwich complex, which was then reacted with 3,3’,5,5’-tetramethyl benzidine solution. The absorbance was measured using a Spark Control multimode microplate reader (Tecan) at 450 nm. The concentration of Aβ in samples was determined from an ELISA calibration curve.

### 4.6. Statistical Analyses

Statistical analyses were performed using GraphPad Prism 7.02 (GraphPad Software; San Diego, CA, USA). The Mann–Whitney U-test and non-repeated ANOVA followed by Dunnett’s multiple comparison test were used for the analysis of significant differences. A value of *p* < 0.05 was considered to indicate a significant difference.

## 5. Conclusions

This study has unveiled the potential of CAC-717 as a novel agent for the degradation/dissociation and inhibition of Aβ aggregates. Given the global burden of Alzheimer’s disease and the prion-like nature of Aβ, our findings suggest that CAC-717 may not only serve as a valuable research tool but also have practical applications in healthcare environments for disinfecting Aβ-contaminated instruments. Future research will focus on evaluating the efficacy of CAC-717 in vivo and its potential integration into existing disinfection and preventive strategies for Alzheimer’s disease and related neurodegenerative conditions.

## Data Availability

The original contributions presented in the study are included in the article, and further inquiries can be directed to the corresponding author.
